# Dietary fat overcomes the protective activity of thrombospondin-1 signaling in the *Apc*^*Min/+*^ model of colon cancer

**DOI:** 10.1038/oncsis.2016.37

**Published:** 2016-05-30

**Authors:** D R Soto-Pantoja, J M Sipes, G Martin-Manso, B Westwood, N L Morris, A Ghosh, N J Emenaker, D D Roberts

**Affiliations:** 1 Department of Cancer Biology, Comprehensive Cancer Center, Wake Forest School of Medicine, Winston-Salem, NC, USA; 2 Department of Surgery, Comprehensive Cancer Center, Wake Forest School of Medicine, Winston-Salem, NC, USA; 3 Laboratory of Pathology, Center for Cancer Research, National Cancer Institute, NIH, Bethesda, MD, USA; 4 Chemical Biology Laboratory, National Laboratory for Cancer Research, Leidos Biomedical Research, Frederick, MD, USA; 5 Nutritional Science Research Group, Division of Cancer Prevention, National Cancer Institute, NIH, Bethesda, MD, USA

## Abstract

Thrombospondin 1 is a glycoprotein that regulates cellular phenotype through interactions with its cellular receptors and extracellular matrix-binding partners. Thrombospondin 1 locally regulates angiogenesis and inflammatory responses that contribute to colorectal carcinogenesis in *Apc*^*Min/+*^ mice. The ability of thrombospondin 1 to regulate responses of cells and tissues to a variety of stresses suggested that loss of thrombospondin 1 may also have broader systemic effects on metabolism to modulate carcinogenesis. *Apc*^*Min/+*^*:Thbs1*^*−/−*^ mice exhibited decreased survival and higher tumor multiplicities in the small and large intestine relative to *Apc*^*Min/+*^ mice when fed a low (5%) fat western diet. However, the protective effect of endogenous thrombospondin 1 was lost when the mice were fed a western diet containing 21% fat. Biochemical profiles of liver tissue identified systemic metabolic changes accompanying the effects of thrombospondin 1 and dietary lipid intake on tumorigenesis. A high-fat western diet differentially regulated elements of amino acid, energy and lipid metabolism in *Apc*^*Min/+*^:*Thbs1*^*−/−*^ mice relative to *Apc*^*Min/+*^*:Thbs1*^*+/+*^mice. Metabolic changes in ketone body and tricarboxylic acid cycle intermediates indicate functional interactions between Apc and thrombospondin 1 signaling that control mitochondrial function. The cumulative diet-dependent differential changes observed in *Apc*^*Min/+*^*:Thbs1*^*−/−*^ versus *Apc*^*Min/+*^ mice include altered amino acid and lipid metabolism, mitochondrial dysfunction, eicosanoids and ketone body formation. This metabolic profile suggests that the protective role of thrombospondin 1 to decrease adenoma formation in *Apc*^*Min/+*^ mice results in part from improved mitochondrial function.

## Introduction

Colon cancer is major public health concern with over 130 000 new cases diagnosed every year and over 50 000 deaths in the United States alone.^1^ While many factors including diet and genetics influence colon cancer progression,^2^ expression of thrombospondin-1 (TSP1) inversely correlates with colon cancer aggressiveness.^3, 4^ TSP1 is a matricellular protein that regulates tissue perfusion, platelet aggregation, angiogenesis, and responses to stress.^4, 5, 6^ Spontaneous tumors can be demonstrated in TSP1 null mice only when they are crossed with other strains that are cancer prone such as the MMTV-Neu model or *p53* null mice.^7, 8^ In several such carcinogenesis models TSP1 expression has been demonstrated to delay premalignant hyperplasia, tumorigenesis, tumor angiogenesis and/or metastasis.^7, 8, 9, 10, 11^

Over 5% of colorectal cancer cases are due to a genetic predisposition, and one frequent abnormality causing predisposition to human colorectal cancer is mutation in the adenomatous polyposis coli (*APC*) gene.^12^ In humans, familial adenomatous polyposis (FAP) is characterized by the onset of multiple adenomas in the colon that can progress to tumors and metastatic forms of colorectal cancer. In mice, heterozygous mutation (*Apc*^*Min/+*^) has generated a clinically relevant model to study colorectal cancer that has provided insights into the onset of sporadic colorectal cancer.^12, 13^ These mice develop many of the characteristics of human FAP patients but have some limitations including aggressivenes of the disease and the formation of most adenomas in the small intestine.^14^ Still many translational applications have been tested successfully in this model including the use NSAID's to prevent or treat colorectal cancer.^12^ The *Apc*^*Min/+*^mouse correspondingly is a widely used model for studying these cancers. To monitor the spontaneous colorectal tumor growth, and the survival of mice exposed to a low fat or western diet, we generated a double transgenic animal harboring the *Apc*^*Min/+*^ locus and lacking TSP1 (*Thbs1*^*−/−*^). Previous studies in the *Apc*^*Min/+*^ murine model of colon cancer demonstrated that loss of TSP1 increases tumor multiplicity in the small and large intestines.^15^ The absence of TSP1 in this model was correlated with an increase in TUNEL positive nuclei in the polyps lacking TSP1. Therefore, the primary role of TSP1 in carcinogenesis in the *Apc*^*Min/+*^ model was attributed to its role inducing apoptosis. On the other hand, recent studies of mice lacking TSP1 or its receptor CD47 have identified important roles in the regulation of lipid and glucose metabolism and the proinflammatory effects of high-fat diets.^16, 17, 18, 19, 20, 21^

In this study, we investigate changes in global liver metabolism associated with the absence of TSP1 in C57BL/6J-ApcMin/J (*Apc*^*Min/+*^) mice, which are highly susceptible to the formation of intestinal adenomas. Liver metabolism responds to changes in metabolites delivered from the intestines and produces metabolites that can influence carcinogenesis in the colon.^22, 23^ As endogenous TSP1 is known to have beneficial effects on colon carcinogenesis in this model in the context of a moderate fat (11%) diet,^15^ carcinogenic and metabolic effects of low- and high-fat diets were assessed in the context of *Thbs1* deletion in this model.

## Results

### Effects of TSP1 on tumor multiplicity in the *Apc*^*Min/+*^ model are modulated by dietary fat

**Figure 1:**
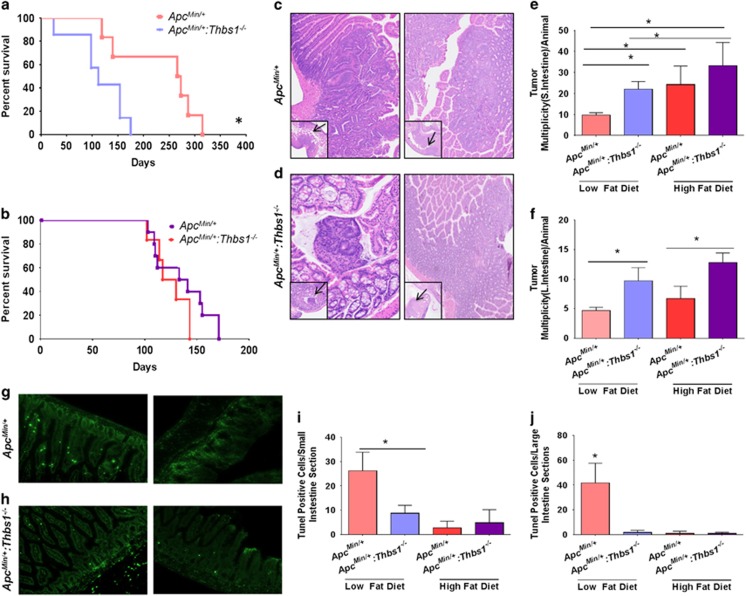
Endogenous thrombospondin-1 limits tumor multiplicity and enhances the survival of *Apc*^*Min/+*^ mice when fed a low-fat diet but not when fed a high-fat diet. WT, *Thbs1*^*−/−*^, *Apc*^*Min/+*^ and *Thbs1*^*−/−*^*:Apc*^*Min/+*^ mice were fed a low-fat (**a**) or a high-fat western diet (**b**) beginning at the time of weaning. (**a**, **b**) Survival was measured over time and evaluated using Log-rank (Mantel–Cox test) and Grehan–Breslow Wilcoxon test. Equal numbers of male and female mice were included (*N*=14) for each group. (**c**, **d**) Small and large intestines were excised at the time of death. **c** and **d** show representative images of lesions (arrows) and quantitation of intestinal lesions. Small intestine (**e**) and large intestine (**f**) lesions were counted under a dissecting microscope and confirmed by H&E staining. *N*=4–5, *P<0.05. (**g**, **h**) Representative images of TUNEL staining in small (left) and large intestine of *Apc*^*Min/+*^ and *Thbs1*^*−/−*^*:Apc*^*Min/+*^ mice quantification is presented for small (**i**) and large intestines (**j**). *N*=6–8 *P<0.05.

Consistent with the previous report that loss of endogenous TSP1 increased polyp formation and progression in *Apc*^*Min/+*^ mice fed a diet that contained 11% fat,^15^ *Apc*^*Min/+*^*:Thbs1*^*−/−*^ mice fed a western diet containing 5% fat had decreased survival relative to *Apc*^*Min/+*^ mice fed the same diet (*P*<0.02, Figures 1a and b). The former completely succumbed by 200 days, whereas 60% of *Apc*^*Min/+*^ mice remained alive at the same time point (*P*<0.03). Feeding a western diet containing 21% fat decreased the survival of *Apc*^*Min/+*^ mice, but the positive effect of endogenous TSP1 on survival was lost in mice fed the high-fat diet (Figure 1b).

Small and large intestines (Figures 1c and d, respectively) were examined under light microscopy to determine whether dietary fat regulates the effects of TSP1 loss on colon carcinogenesis. *Apc*^*Min/+*^*:Thbs1*^*−/−*^ mice that were fed a low-fat diet had a 40% (*P*<0.03) increase in adenoma formation in their small intestine (Figures 1c and e) and a 52% (*P*<0.02) increase in the large intestine when compared with *Apc*^*Min/+*^ mice fed the same diet (Figures 1d and f). Adenoma formation in the small intestine increased in mice of both genotypes fed a high-fat diet. *Apc*^*Min/+*^ mice fed a high-fat diet had a 60% (*P*<0.03) increase in adenoma formation when compared with mice of the same genotype fed a low-fat diet. Moreover, *Apc*^*Min/+*^*:Thbs1*^*−/−*^ mice fed a high-fat diet had 34% (*P*<0.05) increased adenoma formation when compared with mice of the same genotype fed a low-fat diet (Figures 1e and f). When fed a high-fat diet, however, lesion formation in the small intestine was not significantly different between mice of these two genotypes (Figure 1f). More relevant to human APC-dependent colon cancers, *Apc*^*Min/+*^*:Thbs1*^*−/−*^ mice fed a high-fat diet had a 48% (*P*<0.02) increase in adenoma formation in the large intestine when compared with *Apc*^*Min/+*^ mice fed the same diet (Figure 1e). Dietary fat consumption can affect the induction of cell proliferative capacity and death in intestinal tissue.^24^ We assessed cell death in our model by TUNEL staining of tissues (Figures 1g–j). Consumption of a low-fat diet increased TUNEL positive nuclei in *Apc*^*Min/+*^mice to 26% (*P*<0.001) when compared with 8% in *Apc*^*Min/+*^*:Thbs1*^*−/−*^ (Figures 1g). In large intestines we observed a 40% (*P*<0.001) increase in TUNEL positive staining in large intestines of *Apc*^*Min/+*^mice when compared with a 3% increase in *Apc*^*Min/+*^*:Thbs1*^*−/−*^ (Figures 1h). However, induction of cell death was inhibited with the consumption of a high-fat diet in all phenotypes (Figures 1i and j). This implies that consumption of high-fat diet inhibits the activation of pro-apoptotic genes, which may explain the reduced tumor numbers observed in the *Apc*^*Min/+*^ mice. Therefore, a high-fat diet selectively increases adenoma formation in the small intestine, but the protective effects of endogenous TSP1 in the small intestine decrease when dietary fat levels increase.

### TSP1 regulates systemic metabolic responses to a high-fat diet in the *Apc*^*Min/+*^ model

Previous studies of *Apc*^*Min+*^*:Thbs1*^*−/−*^ mice focused on local effects of TSP1 on angiogenesis and inflammatory responses in the intestinal microenvironment where adenomas arise.^15, 25, 26^ However, evidence is accumulating that TSP1 has systemic effects on tissue and metabolic responses to stress, including that caused by a high-fat diet.^6, 17, 18, 19, 27^ In addition to the important local role of Apc in intestinal carcinogenesis, systemic changes in metabolism were recently identified that correlate with high-fat diet-induced risk in the *Apc*^*Min/+*^ model^28, 29^ and patients with adenomatous polyps.^30^ To address whether endogenous TSP1 has systemic effects that could account for its dietary fat-dependent effects on carcinogenesis, we performed a global metabolomics analysis using liver tissue from each genotype of mice fed the low- or high-fat diets. Quantitative data was obtained for 523 named biochemicals, and of these ANOVA analysis indicated that 24% exhibited variation that was mainly attributable to genotype, 37% exhibited variation that was mainly attributable to diet and 31% exhibited variation related to the interaction between diet and genotype (Table 1).

All genotypes showed global variations associated with dietary fat intake, but the absence of TSP1, either in a wild type (WT) *Apc*^*+/+*^ background or in the *Apc*^*Min/+*^ mice, was associated with larger numbers of metabolites that either increased or decreased when the mice were fed a high-fat diet (Table 2). These increases indicated that the presence of TSP1 globally limits the effects of diet to alter liver metabolism.

**Figure 2:**
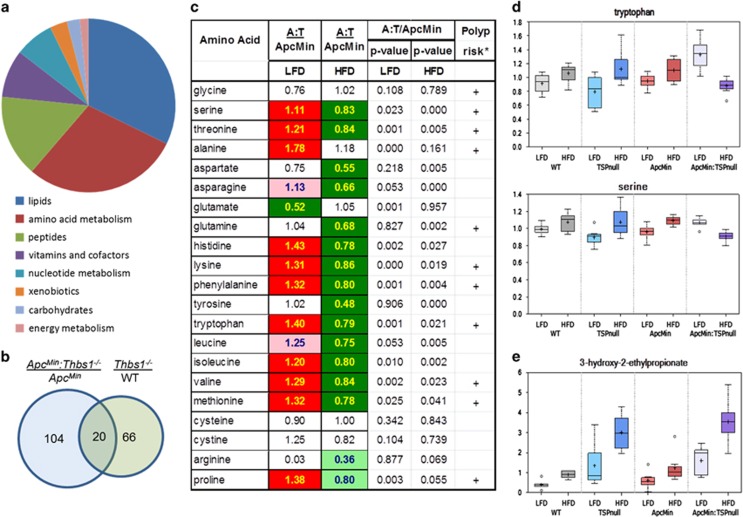
Thrombospondin-1 and Apc regulate amino-acid metabolism. (**a**) Pie chart depicting metabolites that exhibited significant diet-dependent changes in levels in *Thbs1*^*−/−*^*:Apc*^*Min/+*^ mice relative to *Apc*^*Min/+*^ fed the same diets. (**b**) Comparison of metabolites showing differential expression in *Thbs1*^*−/−*^*:Apc*^*Min/+*^ mice versus *Apc*^*Min/+*^ mice and those differing in *Thbs1*^*−/−*^ versus WT mice. (**c**) Relative levels of amino acids in liver of *Thbs1*^*−/−*^*:Apc*^*Min/+*^mice versus *Apc*^*Min/+*^ mice fed low-fat diet or high-fat diet. Polyp risk associated with altered circulating levels of the indicated amino acids (+) is data from Dazard *et al.*^29^ Solid red and green cells indicate *P*<0.05, and shaded cells indicate 0.05<*P*<0.1. (**d**) Levels of representative amino acids (tryptophan, serine) in livers of the indicated mice and diet conditions. (**e**) Levels of the isoleucine catabolite 3-hydroxy-2-ethylpropionate. For the box plots: center horizontal line, median value; +, mean value; box height, limits of upper and lower quartiles; whiskers, max and min of distribution; ○, extreme data point (*N*=7–8).

To identify specific metabolites that could account for this protective activity of TSP1 we focused on metabolites that were differentially altered by high-fat diet in *Apc*^*Min/+*^*:Thbs1*^*−/−*^ mice when compared with *Apc*^*Min/+*^ mice with a WT *Thbs1* locus. Of the named metabolites, 124 showed significant differences in expression between these genotypes that were lost or altered when the mice were fed the high-fat diet (Figure 2a). Comparing liver metabolites from *Thbs1*^*−/−*^ versus WT mice, we found 86 metabolites that were sensitive to dietary fat, and of these only 20 overlapped with those in the *Apc*^*Min+*^*:Thbs1*^*−/−*^ versus *Apc*^*Min+*^ comparison (Figure 2b). Therefore, some of the metabolic changes can be primarily related to the *Thbs1* genotype, but a majority are specific to *Thbs1*^*−/−*^ in the *Apc*^*Min/+*^ context. The majority of the differentially expressed metabolites that were sensitive to dietary fat in *Apc*^*Min+*^*:Thbs1*^*−/−*^ versus *Apc*^*Min/+*^ mice comprised amino acid and lipid metabolites. Eight of the 20 overlap metabolites in Figure 2b were amino-acid metabolites. Most metabolites had higher relative expression in livers of *Apc*^*Min/+*^*:Thbs1*^*−/−*^ mice, which were either lost or reversed in livers from mice fed the high-fat diet. This trend is illustrated in Figure 2c for liver amino-acid levels. Levels of 11 of the 20 amino acids were higher in *Apc*^*Min/+*^*:Thbs1*^*−/−*^ mice than *Apc*^*Min/+*^ mice on the low-fat diet, but 14 of the 20 amino acids were significantly lower in the *Apc*^*Min/+*^*:Thbs1*^*−/−*^ mice when fed the high-fat diet. Notably, 10 of the affected amino acids coincide with amino acids that, when quantified in plasma of *Apc*^*Min/+*^ mice fed high- versus low-fat diet, significantly correlated with adenoma numbers in those mice.^29^ Therefore, we predict that TSP1-dependent changes in liver amino-acid metabolism contribute to the effects of TSP1 to modulate the effect of dietary fat on intestinal carcinogenesis.

Examining two representative amino acids in each of the genotypes reveals that the high-fat diet increased amino-acid levels in WT mice and the two single transgenic strains relative to the respective genotypes fed the low-fat diet (Figure 2d). In contrast, the high-fat diet in the double transgenic *Apc*^*Min/+*^*:Thbs1*^*−/−*^ mice broadly decreased levels of the same amino acids. The affected amino acids include the two exclusively ketogenic amino acids, lysine and leucine, as well as three additional amino acids that are partially ketogenic: isoleucine, tryptophan and phenylalanine. This implicates altered mitochondrial metabolism in the broad effects of TSP1 on amino-acid metabolism. The pattern shown for amino acids extends to most of the 36 amino-acid metabolites that exhibited differential modulation by dietary fat, and most of these are known catabolites of the respective amino acids in Figure 2b (Supplementary Table). In some cases the absence of TSP1 may cause elevation of an amino acid by blocking its catabolism. An illustrative example is shown for isoleucine (Figure 2c). The isoleucine catabolite 3-hydroxy-2-ethylpropionate accumulated in liver of both *Thbs1*^*−/−*^ mouse strains, but was not affected by the *Apc* genotype (Figure 2e).

### Effects of *Thbs1* and *Apc^Min^* on lipid metabolism

**Figure 3:**
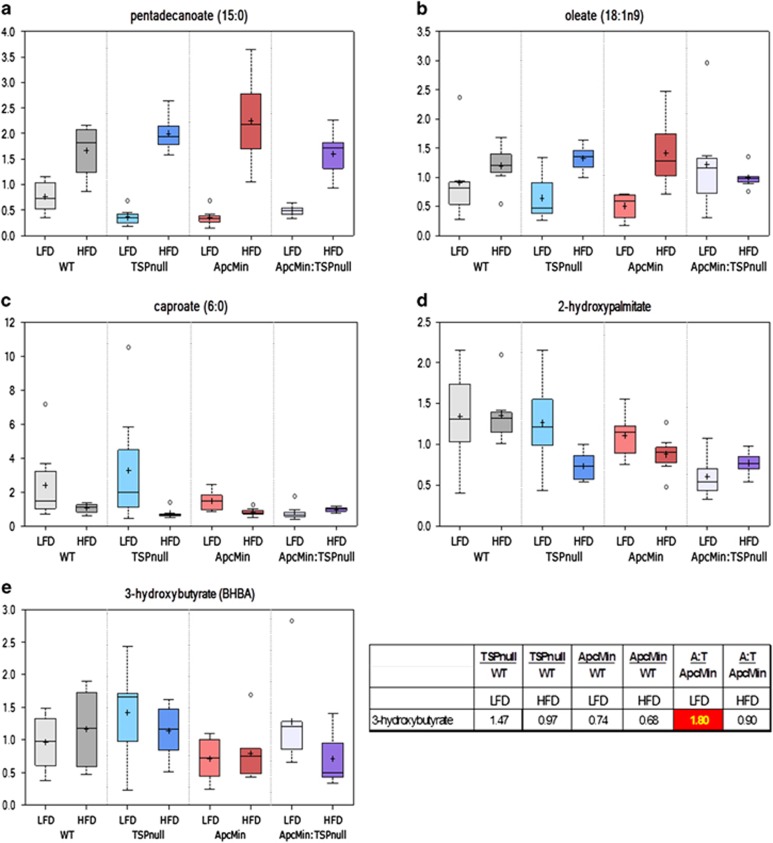
Thrombospondin-1 regulates lipid metabolism in liver tissue of *Apc*^*Min/+*^ mice fed a low-fat diet or a high-fat diet. WT, *Thbs1*^*−/−*^, *Apc*^*Min/+*^ and *Thbs1*^*−/−*^*:Apc*^*Min/+*^ mice were fed a low-fat or a high-fat western diet at the time of weaning. Livers were excised and flash-frozen for metabolomics analysis. (**a**) Representative long-chain free fatty acid derived from dietary fat, pentadecanoic acid, that shows induction by high-fat diet (HFD) independent of genotype, (**b**) levels of oleate acid, representative of fatty acids that are endogenously synthesized and show genotype- and diet-dependence, (**c**) caproate, a short chain fatty acid that exhibits suppression by high-fat diet that is lost in *Thbs1*^*−/−*^*:Apc*^*Min/+*^ background, (**d**) representative hydroylated fatty acid that shows differential regulation in *Thbs1*^*−/−*^*:Apc*^*Min/+*^ versus *Apc*^*Min/+*^ mice, and (**e**) the ketone body metabolite 3-hydroxybutyrate is selectively elevated in *Thbs1*^*−/−*^*:Apc*^*Min/+*^ mice fed a low-fat diet. *N*=8.

Consistent with their increased fat consumption, all strains of mice fed the high-fat diet showed the expected hepatic accumulation of various medium-chain and long-chain saturated, monounsaturated and polyunsaturated free fatty acids (Supplementary Table). Elevations in free fatty acids were similar across groups (for example, pentadecanoic acid Figure 3a). Pentadecanoic acid is abundant in butterfat and is a documented metabolic marker for animals fed this diet^31^ Increases in select medium-chain free fatty acids were more pronounced in the *Apc*^*Min/+*^*:Thbs1*^*−/−*^ samples (Supplementary Table). From the analysis in Figure 2a, 40 of the named lipid metabolites showed differential regulation by high-fat diet in *Apc*^*Min/+*^*:Thbs1*^*−/−*^ mice versus *Apc*^*Min/+*^ mice. Among these, long chain lipids such as oleate generally showed increased levels with high-fat diet that were lost in the *Apc*^*Min/+*^*:Thbs1*^*−/−*^ mice (Figure 3b). Conversely, short-chain lipids such as caproate and seven of the detected hydroxy-fatty acids (for example, 2-hydroxypalmitate) showed decreases with the high-fat diet that were absent in the *Apc*^*Min/+*^*:Thbs1*^*−/−*^ mice (Figures 3c and d). Therefore, crosstalk between *Thbs1* and *Apc^Min^* selectively regulates metabolism of a subset of lipids.

**Figure 4:**
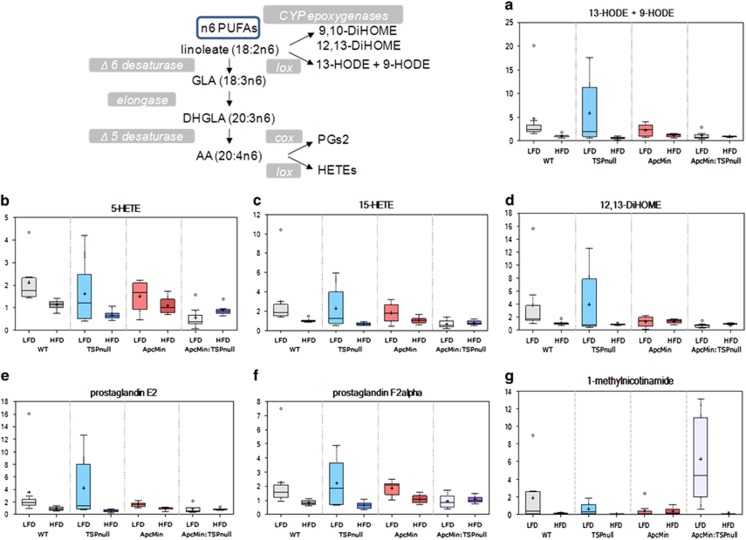
Thrombospondin-1 synergizes with dietary fat to limit liver inflammatory eicosanoid metabolism in *Apc*^*+/+*^ but not in *Apc*^*Min/+*^ mice. Eicosanoid and 1-methylnicotinamide levels were determined in liver tissue isolated from WT, *Thbs1*^*−/−*^, *Apc*^*Min/+*^ and *Thbs1*^*−/−*^*:Apc*^*Min/+*^ mice fed a low fat or a high fat western diet at the time of weaning. (**a**) 13- Hydroxyoctadecadienoic acid (13-HODE) and 9-hydroxyoctadecadienoic acid (9-HODE) are isobaric, and cannot be distinguished by selective ion monitoring. (**b**, **c**) 5-Hydroxyicosatetraenoic acid (5-HETE) is a metabolite of arachidonic acid produced by 5-lipoxygenase (lox), and 15-hydroxyicosatetraenoic acid (15-HETE) is produced by 15-lipoxygenase-1. (**d**) 12,13-Dihydroxy-9Z-octadecenoic acid (12,13-diHOME) is a cytochrome P450 epoxygenase (CYP-epoxygenase) metabolite of linoleate. (**e**, **f**) Prostaglandins E2 and F2α are bioactive prostaglandins derived from the cyclooxygenase (COX) product PGH2. (**g**) 1-Methylnicotinamide is an anti-inflammatory COX-dependent nicotinamide catabolite.

Intake of a high-fat diet was associated with hepatic accumulation of fatty acids, which can affect cell energy metabolism and signaling to stimulate carcinogenesis. Both fatty acid oxidation and the observed increases in ketogenic amino acids provide sources of acetyl-CoA. *Apc*^*Min/+*^*:Thbs1*^*−/−*^ mice fed the low-fat diet had 1.8-fold elevated levels relative to *Apc*^*Min/+*^ mice of the ketone body 3-hydroxybutyrate (BHBA, *P*=0.02, Figure 3e). This elevation was lost in *Apc*^*Min/+*^*:Thbs1*^*−/−*^ mice fed a high-fat diet, representing a significant reduction (*P*=0.04, Figure 3e). BHBA is synthesized in the liver from excess acetyl-CoA and utilized by extra hepatic tissues to meet energy demands during periods of starvation. BHBA has been identified as a serum biomarker of colorectal carcinoma and esophageal carcinoma.^32, 33^ Lipid metabolites involved in eicosanoid biosynthesis showed strong dependences on dietary fat as well as TSP1 (Figure 4). The *Apc*^*Min/+*^ mutant is known to increase COX2 expression and consequently prostaglandin PGE2 levels via a CtBP1-dependent mechanism,^34, 35, 36^ and COX2 is known to regulate TSP1 expression,^37, 38^ but no change in PGE2 expression was reported in *Thbs1*^*−/−*^ macrophages.^39^ However, *Thbs1*^*−/−*^ liver showed strong elevation in PGE2 levels as well as in PGF2 and 5- and 15-hydroxyeicosatetraenoic acid levels (Figures 4b, c, e and f). This indicates a general inhibitory effect of TSP1 on eicosanoid biosynthesis from n6-polyunsaturated fatty acids. Notably, the high-fat diet suppressed these elevations in *Thbs1*^*−/−*^ liver. The high-fat diet was also inhibitory in the other genotypes, except for *Apc*^*Min/+*^*:Thbs1*^*−/−*^ mice where levels were low in mice fed both diets.

Levels of a major nicotinamide catabolite that is produced in liver were consistent with the observed suppression of eicosanoids in the *Apc*^*Min+*^*:Thbs1*^*−/−*^ mice. 1-Methylnicotinamide is a potent anti-inflammatory and antithrombotic molecule, and its activity was blocked by a COX2 inhibitor.^40, 41^ Although expression of its biosynthetic enzyme nicotinamide-*N*-methyltransferase was induced in some obesity models,^42^ 1-methylnicotinamide levels in WT and *Thbs1*^*−/−*^ liver were suppressed by high fat intake and were basally lower in *Apc*^*Min/+*^: mice (Figure 4g). Remarkably, 1-methylnicotinamide levels were strongly elevated in the *Apc*^*Min/+*^*:Thbs1*^*−/−*^ mouse liver, but it was also suppressed by feeding the high-fat diet. In contrast, further catabolism of 1-methylnicotinamide to N1-methyl-2-pyridone-5-carboxamide was significantly decreased in *Apc*^*Min/+*^*:Thbs1*^*−/−*^ mice relative to *Apc*^*Min/+*^ mice (Supplementary Table). The proximal target of 1-methylnicotinamide remains unclear, so further study will be required to determine whether it is responsible for the observed suppression of eicosanoids in *Apc*^*Min/+*^*:Thbs1*^*−/−*^ mice.

### TSP1 regulates mitochondrial energy metabolism in *Apc*^*Min/+*^mice

**Figure 5:**
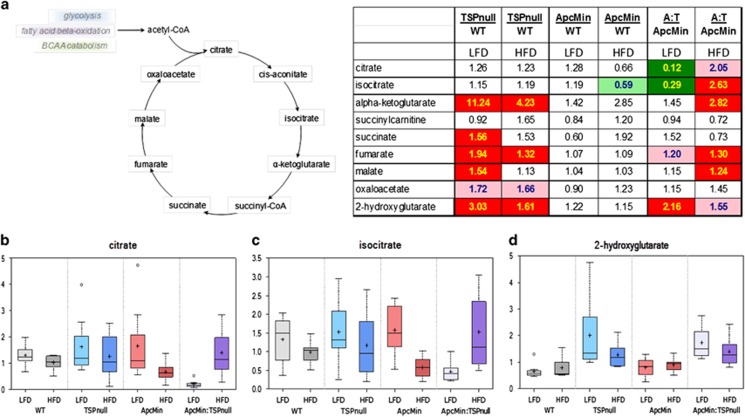
Thrombospondin-1 and dietary fat regulate TCA cycle metabolites and 2-hydroxyglutarate in *Apc*^*Min/+*^ mice. WT, *Thbs1*^*−/−*^, *Apc*^*Min/+*^ and *Thbs1*^*−/−*^*:Apc*^*Min/+*^ mice were fed a low-fat or a high-fat western diet at the time of weaning. Livers were excised and flash frozen for metabolomics analysis. (**a**) Citric acid cycle metabolites and ratios for the indicated metabolites in livers from the respective mice fed low- or high-fat diets. Solid red and green cells indicate *P*<0.05, and shaded cells indicate 0.05<*P*<0.1. (**b**) citrate, (**c**) isocitrate, (**d**) 2-hydroxyglutarate levels in livers from mice of the indicated genotypes, *N*=8.

The global changes in ketogenic amino acid and lipid metabolites are consistent with regulation of mitochondrial metabolism by TSP1. We recently reported that loss of the TSP1 receptor CD47 regulates metabolic flux through the TCA cycle and altered citrate levels by regulation of citrate synthase activity.^21^ Two TCA cycle intermediates, citrate and isocitrate, showed differential diet-dependent regulation in *Apc*^*Min/+*^*:Thbs1*^*−/−*^ mice versus *Apc*^*Min/+*^ mice (Figures 5a–c). A comparison between *Apc*^*Min/+*^*:Thbs1*^*−/−*^ and *Thbs1*^*−/−*^ mice fed the low-fat diet showed more extensive suppression of downstream metabolites in the TCA cycle (Figure 5a). These differences may be attributable to the Apc^Min/+^ phenotype and were lost when with a high fat intake. These downstream effects of Apc^Min/+^ on TCA metabolites are consistent with a previous analysis of SW480 colon carcinoma cells expressing mutant Apc that identified succinate, fumarate and malate as Apc-sensitive metabolites.^28^ However, no significant differences in TCA metabolites were observed when comparing *Apc*^*Min/+*^ mice with WT mice. In contrast, *Thbs1*^*−/−*^ mouse livers showed significant elevations in α-ketoglutarate, succinate, fumarate and malate levels relative to WT mice. High-fat diet decreased these elevations, but α-ketoglutarate and fumarate remained significantly elevated.

Elevation of the oncogenic metabolite 2-hydroxyglutarate (2HG) is associated with several cancers, and 2HG drives progression of these cancers by epigenetic reprogramming.^43, 44^ Elevated 2HG levels in cancer can be caused by mutations in isocitrate dehydrogenase-2 (IDH2) in the TCA cycle,^43^ loss of L-2HG dehydrogenase^45^ or by increased IDH2 expression induced by cMyc.^46^ 2HG increases malignant progression by several mechanisms including increasing cancer stem cell characteristics.^46^ 2HG could, therefore, be relevant to the effects of TSP1 in the Apc^Min^ model because tissues in *Thbs1*^*−/−*^ mice express elevated cMyc and other stem cell characteristics.^47^ The *Apc*^*Min/+*^ mice showed no changes in 2HG relative to WT mice, but 2HG levels were significantly higher in *Thbs1*^*−/−*^ mice on both diets (Figures 5a and d). Levels were also significantly elevated in *Apc*^*Min/+*^*:Thbs1*^*−/−*^ mice versus *Apc*^*Min/+*^ mice fed a low-fat diet, but when fed a high-fat diet the significant elevation was lost. This correlates with the selective protective role of TSP1 in the Apc^Min^ model that was seen only in the low-fat diet. A causative role for 2HG in this effect of TSP1 expression on intestinal carcinogenesis remains to be determined.

## Discussion

A global metabolic profiling study revealed significant basal, genotype-specific and dietary fat-dependent metabolic differences in liver tissue of WT versus *Thbs1*^*−/−*^ and *Apc*^*Min/+*^ mice. Analysis of liver tissue from the double transgenic *Apc*^*Min/+*^*:Thbs1*^*−/−*^ mice revealed additional metabolic changes that could not be predicted based on those of the two parental transgenic strains. When fed a low-fat diet *Apc*^*Min/+*^*:Thbs1*^*−/−*^ mice exhibited a number of metabolic alterations relative to *Apc*^*Min/+*^*:Thbs1*^*+/+*^ mice that were lost with high-fat intake. A majority of these involved amino acid, eicosanoid and lipid metabolism. Ketogenic amino acids and lipid metabolism changes were associated with dysregulation of TCA cycle metabolites that suggest mitochondrial dysfunction in livers of the *Apc*^*Min/+*^*:Thbs1*^*−/−*^ mice. The dysregulation of cell energy metabolites was associated with increased tumor multiplicity and decreased survival of *Apc*^*Min/+*^*:Thbs1*^*−/−*^ mice when fed a low-fat diet. The increase in tumor multiplicity was observed in both the small and large intestine.

The *Apc*^*Min/+*^ model is a clinically relevant tumor model of FAP and spontaneous colon cancer. However one of the limitations of this model is that most lesions are observed in the small intestines, which does not mirror the human disease. Deletion of TSP1 caused an increased in colonic lesions independent of diet when compared with *Apc*^*Min/+*^, which may be a more clinically relevant model to study colon cancer as it mimics the observed loss of TSP1 and the phenotype associated with it in humans. Other genetic deletions in the *Apc*^*Min/+*^ mouse have led to increased lesions. Absence of glutathione transferase gene in the *Apc*^*Min/+*^ mouse led to six-fold increase in colon lesions.^48^ This was attributed in part to increase in pro-inflammatory signaling and regulation of iNOS. The *Apc*^*Min/+*^*:Thbs1*^*−/−*^ mice also showed regulation of inflammatory signaling and showed regulation of reduced glutathione and oxidized glutathione in livers, which could explain the increased lesions in the large intestine in both models.

Several studies have demonstrated that loss or inhibition of TSP1 expression increases colon carcinogenesis and malignant progression.^49, 50, 51, 52^ Decreased TSP1 expression in adenomas of *Apc*^*Min/+*^ mice was associated with a reduced apoptotic index, increase in proliferation and differential patterns of vessel density.^15^ *Apc*^*Min/+*^*:Thbs1*^*−/−*^ showed increased vascularization in pre-malignant intestinal tissue but no significant differences in vascularization between adenomas and carcinomas between *Apc*^*Min/+*^ and *Apc*^*Min/+*^*:Thbs1*^*−/−*^. This also correlated with no differences in expression of VEGF, which led to the conclusion that TSP1 may regulate the initial stages of carcinogenesis when neovascularization to sustain a tumor lesion is low. Correspondingly, once tumors became well established, the effect of TSP1 deletion was decreased . However, our studies indicate that TSP1 may also be involved in later stages of colon carcinogenesis because *Apc*^*Min/+*^ animals that were fed a low-fat diet, which can decrease colon cancer formation, showed decreased survival in the absence of TSP1 expression. In the context of a high-fat diet, the absence of TSP1 led to multiple metabolic alterations in livers of 12-week-old mice that could explain late-stage systemic effects of TSP1 on colon cancer progression.

Our study indicates that an increase in TCA cycle metabolites could be due to the increased glycolytic flux, which is characteristic of colon cancer.^53^ Consistent with this hypothesis, loss of the TSP1 receptor CD47 altered both glycolytic and TCA cycle flux in a human T-cell line and *cd47*^*−/−*^ mice.^21^ However, the present results suggest decreased TCA cycle activity and oxidative phosphorylation. As both β-oxidation and ketogenesis occur in the mitochondria, and medium-chain free fatty acids can freely cross mitochondrial membranes to facilitate β-oxidation, one explanation for the cumulative differential changes observed in *Apc*^*Min/+*^*:Thbs1*^*−/−*^ mice fed a high-fat diet may involve mitochondrial dysfunction, resulting in decreased fatty acid oxidation and ketone body formation, along with the accumulation of free fatty acids. Perturbations in the TCA cycle intermediates and biochemicals related to lipid metabolism are indicative of changes in fatty acid handling and utilization in high-fat diet-fed *Apc*^*Min/+*^*:Thbs1*^*−/−*^ mice as compared with the other study groups. Cancer cells use amino acids as energy sources or to generate metabolites to fuel cell proliferation and growth.^54^ We observed that amino-acid metabolite levels were increased in *Apc*^*Min/+*^*:Thbs1*^*−/−*^ fed a low-fat diet when compared with *Apc*^*Min/+*^ alone. This could contribute to the differences in tumor multiplicity and survival within this group, as other studies where serum metabolites were analyzed found increased amino-acid metabolites in *Apc*^*Min/+*^ mice as well as in the serum of mice bearing colon tumors of the HT-29 cell line where the *APC* gene was truncated.^28^ On the other hand, some amino-acid metabolite levels were depleted in the *Apc*^*Min/+*^*:Thbs1*^*−/−*^ fed a high-fat diet when compared with *Apc*^*Min/+*^ mice. This is a less surprising finding as this could indicate that the low levels of amino-acid metabolites are due in part to increased uptake to fuel cell growth. Lower liver levels of ketogenic amino acids and metabolites may reflect higher mitochondrial β-oxidation in mice fed the high-fat diet. Consistent with this hypothesis, amino-acid levels were depleted by high fat intake in drosophila.^55^ This was attributed to increased levels of urea and uric acid affecting nitrogen metabolism and the increased analplerotic substrates in the TCA cycle. In circumstances where fat is the main source of energy, lipids cannot readily enter the TCA cycle via anaplerotic pathways to generate acetyl-CoA.^55^ In this situation, levels of acetyl-CoA are generated via β-oxidation. Therefore, amino-acid metabolism can serve as a primary source of anaplerotic substrates to sustain the TCA cycle and become depleted during instances of low glucose utilization.^55^ Another point of regulation of the TCA cycle in our models is indicated by the levels of 3-hydroxybutyrate in *Apc*^*Min/+*^*:Thbs1*^*−/−*^ mice. 3-Hydroxybutyrate is generated in the liver from acetyl-CoA during periods of low glucose levels or starvation. This should occur in the context of a low-fat diet that is not deficient in carbohydrate sources, thus indicating a shift in glucose utilization to ketone body formation as a source of energy may be driven by the absence of TSP1 in the *Apc*^*Min/+*^ mice. During starvation, ketone bodies are used as an energy source, and the decreased use of glucose by peripheral organs such as the liver suggest an increased dependence on fat as an energy source.^56^ Since *Apc*^*Min/+*^*:Thbs1*^*−/−*^ have increased tumor multiplicity, glucose utilization may shift toward tumors while leaving peripheral organs to rely on fatty acid and ketone body formation to maintain energy levels. This shift in ketone body and glucose utilization due to starvation can increase pathways such as autophagy that cause increased carcinogenesis. Autophagy is demonstrated to be activated in murine intestinal epithelium and human colon cancer.^57, 58^ In an *Apc*^*Min/+*^ model, deletion of autophagy-related protein-7 (*Atg7*) led to AMPK activation and reduction in carcinogenesis.^57^ Loss of the TSP1/CD47 signaling axis is implicated in the activation autophagy in non-transformed tissue.^59^ Although the role of TSP1/CD47 signaling in regulation of autophagy in cancer is unknown, circulating levels of TSP1 may inhibit this pathway to ameliorate cancer development.

Moreover, *Apc*^*Min/+*^*:Thbs1*^*−/−*^ *mice* fed a low-fat diet show elevated levels of 2HG, which is known as an oncometabolite.^60^ Several factors including mutations in isocitrate dehydrogenase lead to accumulation of 2HG, which is considered a poor prognostic factor in many cancers including colon cancer.^61, 62^ The accumulation of this metabolite is another indication of defects in metabolism that are caused by the deletion of *Thbs1* in the *Apc*^*Min/+*^ mice and could lead to more aggressive formation of tumors in this model.

The negative effects of endogenous TSP1 on PGE2 levels in mice fed the low-fat diet may also contribute to the delay of carcinogenesis by endogenous TSP1. The loss of PGE2 suppression by TSP1 in mice fed the high-fat diet is consistent with our finding that the high-fat diet suppressed the protective effects of TSP1 in *Apc*^*Min/+*^ mice. COX2 expression is elevated in polyps of *Apc*^*Min/+*^ mice^34^ and in human colorectal cancer.^63^ Apc^Min^ is defective in regulation of COX2 via inhibition of CtBP1.^36^ COX2 inhibitors are well documented by epidemiological studies to delay colon carcinogenesis.^64, 65^ Thus, our data indicates that TSP1, Apc and dietary fat are convergent regulators of PGE2 levels. *Thbs1*^*−/−*^ mice have elevated liver levels of multiple eicosanoid metabolites including 9-HODE, 13-HODE and 12,13-diHOME, which suggest that the regulation of PGE2 levels by TSP1 occurs upstream at least at the level of linoleate availability. The proximal molecular targets of TSP1 signaling in the eicosanoid pathway remain to be determined.

Studies have demonstrated that a low-fat diet can prevent colon tumor formation, but the relative risk associated with dietary fat in humans remains unclear.^66, 67^ Our study demonstrates a protective effect of endogenous TSP1 that is sensitive to dietary fat intake. The loss of TSP1 expression that has been documented to result from loss of tumor suppresser genes and activation of oncogenes associated with colon carcinogenesis could contribute to promoting early and late stages of colon tumor formation.^4^ Thus, *Apc*^*Min/+*^*:Thbs1*^*−/−*^ mice could be useful to identify TSP1-independent effects of cancer genes on cancer progression. Another advantage of the *Apc*^*Min/+*^*:Thbs1*^*−/−*^ model is that more tumors form in the large intestine when compared with the *Apc*^*Min/+*^ mice. Therefore, this model may be more clinically relevant to study the role of APC in human colon carcinogenesis and responses to treatment.

## Materials and methods

### Apc^Min^ model of colorectal cancer

Experimental protocols, housing and care of mice were conducted in an AAALAC-approved facility according to the animal study protocols LP-026, approved by the National Cancer Institute Animal Care and Use Committee, and ASP 12-461 approved by the NCI-Frederick Animal Care and Use Committee. WT, *Apc*^*Min/+*^ mice and *Thbs1*^*−/−*^ mice on a C57Bl/6 background were purchased from Jackson Laboratories. All mice were maintained and bred at least two generations on a low fat western diet (AIN-76A containing 5%. at, Research Diets) to equalize dietary effects on their epigenetic context and microbiomes. *Apc*^*Min/+*^ mice were crossed with *Thbs1*^*−/−*^ mice to generate the *Thbs1+/-*:*Apc*^*Min/+*^ strain, and these mice were crossed with *Thbs1*^*−/−*^ mice to generate *Thbs1*^*−/−*^:*Apc^Min/^* mice. Mice were genotyped prior to weaning and randomized by stratification keeping equal sample sizes exposed to diets during the same period of time.

The *Apc* locus was genotyped using allele-specific SNP PCR. Briefly mouse tail genomic DNA was extracted using Puregene Core Kit A (part number 1042601). Twenty nanograms of DNA was added to 1 × TaqMan Genotyping Master Mix (Applied Biosystems Part number 4371355) containing the following Custom TaqMan SNP Genotyping Assay primers: forward

AHD16ZB_F 5′-GGGAAGTTTAGACAGTTCTCGTTCT-3′ and reverse AHD16ZB_R 5′-TAAGCACTGAGGCCAATACCT-3′ and the minor groove-binder probes AHD16ZB_V VIC dye-5′-CTCTCTCCAAACTTCT-3′-quencher and AHD16ZB_M FAM dye-5′-TCTCTCTCCTAACTTCT-3′-quencher. PCR amplification was performed using an Applied Biosystem ViiA 7 Real-Time PCR System: 60 °C for 30 s, 95 °C for 10 min followed by 40 cycles of 92 °C for 15 s and 60 °C for 1 min. The *Thbs1* locus was genotyped using PCR primers as described.^68^

Mice were pair-fed a low fat (AIN-76A 5% fat) or a 21% high-fat western diet (#D12079B, Research Diets) beginning at weaning. The two diets contained equal percentages of protein by weight in the form of casein and equal contents of vitamin and mineral supplements. Basal 5% fat in both diets was provided by corn oil, and the high-fat diet was supplemented to 21% with anhydrous milk fat. Carbohydrates provided by cornstarch, maltodextrin and sucrose were adjusted to yield similar caloric content (3902 kcal% in AIN76A and 4686 kcal% in D12079B). One set of mice was killed at 12 weeks for metabolic and histologic analyses. Livers were harvested between 1300 hours and 1500 hours to control for circadian changes in metabolism and immediately frozen for metabolomics analysis. Additional groups of mice were maintained on low- or high-fat diets until they reached humane endpoints for survival analysis.

### Liver tissue metabolomics

Livers of pair-fed, 12-week-old mice of the described genotypes were extracted, and weighted and equal amounts of liver tissue were flash frozen and submitted to Metabolon, Inc for extraction and analysis as shown previously.^21^ Briefly, the liquid chromatography/mass spectrometry portion of the platform was based on a Waters ACQUITY ultra-performance liquid chromatography and a Thermo Scientific Q-Exactive high resolution/accurate mass spectrometer interfaced with a heated electrospray ionization (HESI-II) source and Orbitrap mass analyzer operated at 35,000 mass resolution.

### Histology

Small and large intestines were collected from mice at 12 weeks and at the time of death. Tissues were fixed in 10% buffered formalin and subsequently sent for paraffin embedding. Tissues were stained with hematoxylin and eosin to determine the structure and were examined under light microscopy. TUNEL staining was performed as described previously.^59^ Intestinal lesions were counted blindly by a pathologist from the Laboratory of Pathology at the National Cancer Institute in five fields at a magnification of × 20.

### Statistical analysis

Missing values were assumed to be below the level of detection. Biochemicals that were detected in all the samples from one or more groups, but not in samples from other groups were assumed to be near the lower limit of detection in the groups in which they were not detected. In this case, the lowest detected level of these biochemicals was imputed for samples in which that biochemical was not detected. Following log transformation and imputation with minimum observed values for each compound, data were protein normalized by Bradford protein assay, and both an ANOVA contrast and two-way ANOVA with random effects were used to identify biochemicals that differed significantly between experimental groups. An estimate of the false discovery rate (*q*-value) was calculated to take into account the multiple comparisons that normally occur in metabolomic-based studies. Application of principal component analysis was used to determine separation of study groups (*N*=8 WT, TSP1 null, *Apc*^*Min/+*^and *N*=7 *Apc*^*Min/+*^*:Thbs1*^*−/−*^). Pathways were assigned for each metabolite, allowing examination of overrepresented pathways. Survival of mice was measured over time and evaluated using log-rank (Mantel–Cox test and Grehan–Breslow–Wilcoxon test, *N*=14).

## Figures and Tables

**Table 1:** Statistical summary of liver metabolites significantly associated with effects of genotype, diet, or the interaction between diet and genotype based on two-way ANOVA analysis (*P*<0.05)

	*Genotype*	*Diet*	*Genotype:diet*
	*Main effect*	*Main effect*	*Interaction*
Significantly altered biochemicals	123	195	164

From analysis of the data set, total of 523 named biochemicals were detected.

**Table 2:** Biochemicals quantified in livers from mice of the indicated genotypes that were significantly altered by feeding HFD versus LFD

	Total (P⩽*0.05*)	*Increased with HFD*	*Decreased with HFD*
WT	91	41	50
*Thbs1 null*	155	83	72
*Apc*^*Min/+*^	97	64	33
*Apc*^*Min/+*^*:Thbs1 null*	177	76	101

Abbreviations: HFD, high-fat diet; LFD, low-fat diet.

Data are from ANOVA contrast analysis of the dataset total of 523 named biochemicals detected.
