# Supplementary figures and images for: Dietary fat overcomes the protective activity of thrombospondin-1 signaling in the ApcMin/+ model of colon cancer
Source: Oncogenesis. 2016 May 30;5(5):e230–. doi: 10.1038/oncsis.2016.37 (PMC4945754; doi:10.1038/oncsis.2016.37)

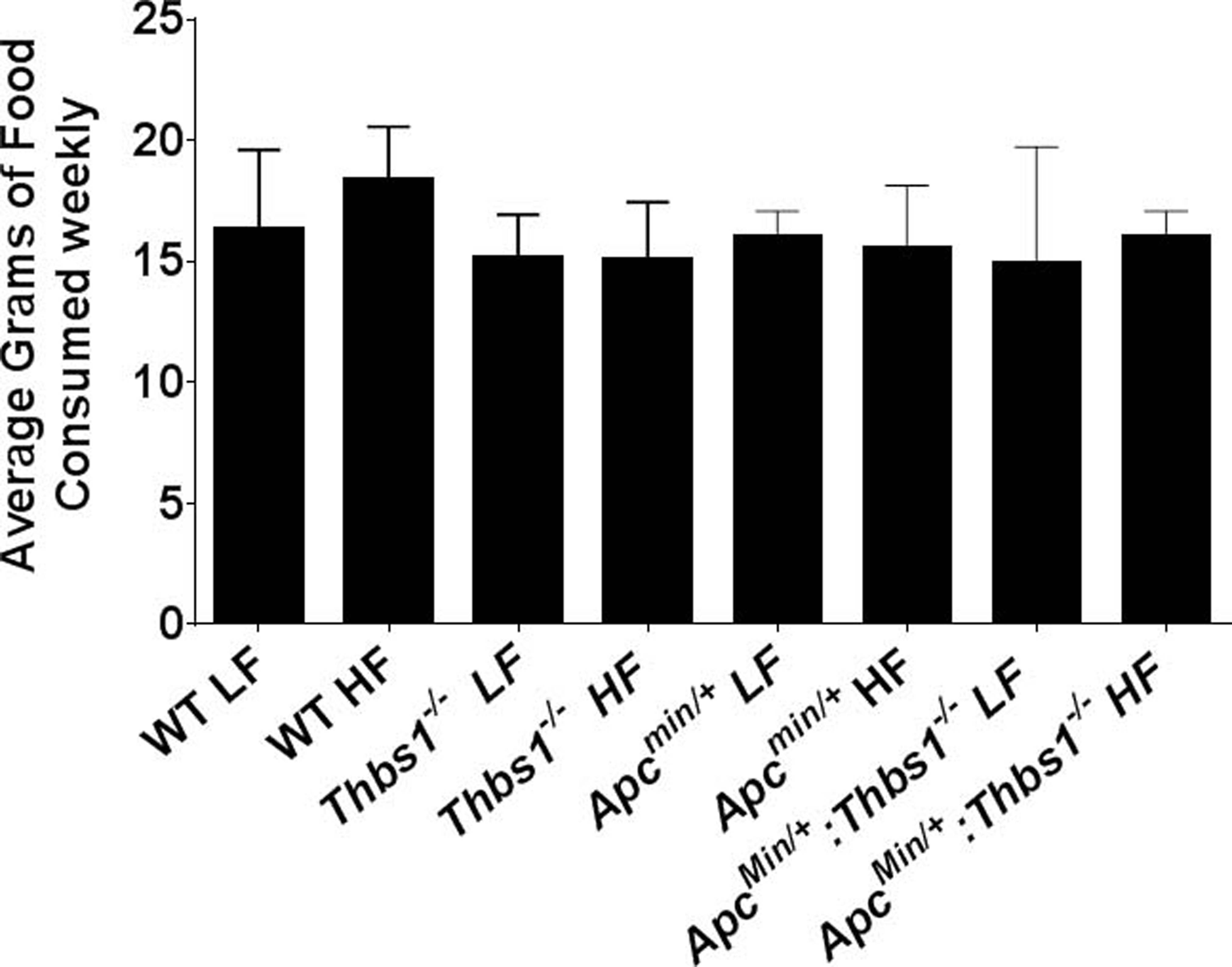

Supplement: Supplementary Figure 1 [file oncsis201637x1.tif]
