# Supplementary material for: Dietary fat overcomes the protective activity of thrombospondin-1 signaling in the ApcMin/+ model of colon cancer
Source: Oncogenesis. 2016 May 30;5(5):e230–. doi: 10.1038/oncsis.2016.37 (PMC4945754; doi:10.1038/oncsis.2016.37)
Supplement: Supplementary Information [file oncsis201637x3.doc]

**Supplemental Table.** In vivo metabolomics data. The first three Excel spreadsheets contain raw, scaled, and normalized data for all liver metabolites analyzed in WT, *ApcMin/+*, *Thbs1-/-*, and *Thbs1-/-:ApcMin/+* mice fed a low fat or a high fat western diet at the time of weaning. The fourth spreadsheet contains the pathway heat map and complete statistical analysis analyzing effects of genotype, radiation, and time on each named metabolite. Dark red cells indicate increased levels with p < 0.05, light red indicates increased levels with 0.05 < p < 0.1. Dark green cells indicate decreased levels with p < 0.05, light green indicates decreased levels with 0.05 < p <0.1. Blue cells indicate ANOVA effects with p < 0.05.

**Supplemental Figure 1.** Average food consumption. Diets for mice of all genotypes where weighted and equal amounts were given in cages housing 4 mice after weaning. Difference in food weight was measured weekly to calculate the average of grams of food consumed per genotype (N=8). One way ANOVA indicates no significant differences in food consumption p>0.05.
